# Time-Interval-Based Collision Detection for 4WIS Mobile Robots in Human-Shared Indoor Environments

**DOI:** 10.3390/s25030890

**Published:** 2025-01-31

**Authors:** Seungmin Kim, Hyunseo Jang, Jiseung Ha, Daekug Lee, Yeongho Ha, Youngeun Song

**Affiliations:** 1 Department of Autonomous Mobility, Korea University, Sejong 2511, Republic of Korea; songarak27@korea.ac.kr (S.K.); kelvin926@korea.ac.kr (H.J.); hajs1234@korea.ac.kr (J.H.); daeku-glee@korea.ac.kr (D.L.); 2 Mobile Robotics Research and Development Center, FieldRo Co., Ltd., Sejong 2511, Republic of Korea; yeongho@fieldro.tech

**Keywords:** 4WIS mobile robot, kinematic modeling, human collision detection, parallel mode, LiDAR, Kalman filter

## Abstract

The recent growth in e-commerce has significantly increased the demand for indoor delivery solutions, highlighting challenges in last-mile delivery. This study presents a time-interval-based collision detection method for Four-Wheel Independent Steering (4WIS) mobile robots operating in human-shared indoor environments, where traditional path following algorithms often create unpredictable movements. By integrating kinematic-based robot trajectory calculation with LiDAR-based human detection and Kalman filter-based prediction, our system enables more natural robot–human interactions. Experimental results demonstrate that our parallel driving mode achieves superior human detection performance compared to conventional Ackermann steering, particularly during cornering and high-speed operations. The proposed method’s effectiveness is validated through comprehensive experiments in realistic indoor scenarios, showing its potential for improving the efficiency and safety of indoor autonomous navigation systems.

## 1. Introduction

The rapid growth of e-commerce and increasing urbanization have intensified challenges in urban transportation systems, particularly in last-mile delivery [1,2]. As online customers demand more frequent and faster deliveries, the concentration of delivery services in urban areas has led to increased pollution, congestion, and operational costs [3,4,5]. Last-mile delivery, accounting for 40% of total supply chain costs, represents the most inefficient segment of logistics services [6]. Many researchers have explored innovative solutions to improve last-mile delivery efficiency [7], with autonomous mobile robots emerging as a promising technology [8,9]. These systems aim to address key challenges in sustainability, customer service, and cost reduction. While aerial delivery systems like drones offer speed advantages, they are limited in carrying capacity [10]. Ground-based mobile robots present a more practical solution for indoor environments, capable of handling multiple parcels per delivery [11,12].

In this study, we utilize a 4WIS mobile robot that addresses several limitations of conventional systems. Traditional mobile robots with fixed or car-like steering mechanisms suffer from restricted maneuverability in confined spaces, unstable sensor orientation during turns, and inefficient trajectory adjustments in dynamic environments [13]. To overcome these limitations, our 4WIS configuration enables enhanced maneuverability through independently controlled wheels. Each wheel’s individual actuation allows precise trajectory adjustments and stable sensor orientation, which is particularly beneficial for indoor navigation [14,15,16].

In order to utilize 4WIS mobile robots in various environments, extensive research on driving modes has been conducted [17,18]. The unique capability of 4WIS robots to steer each wheel independently has led to the development of diverse driving modes, each with distinct characteristics. To provide adequate controllability, researchers have designed a four-mode steering strategy: Ackermann steering, Parallel steering, Crab steering, and Spinning. Ackermann steering, the most commonly used type in mobile robots, mimics car-like systems [19,20,21]. It allows for smooth turns by angling the wheels differently, making it ideal for traditional navigation scenarios. Parallel steering, on the other hand, aligns all wheels in the same direction, enabling sideways movement without changing the robot’s orientation, a valuable feature in tight spaces [22,23].

While each of these modes offers unique advantages, their effectiveness varies depending on the specific environment and task. For indoor environments, Parallel mode demonstrates particular promise due to its ability to maintain stable sensor orientation while maneuvering in confined spaces. However, the implementation of effective navigation strategies in indoor environments presents additional challenges, particularly in collision avoidance.

Traditional reactive collision avoidance behaviors often result in sudden movements and unnecessary path adjustments by robots when sharing spaces with humans. These abrupt changes in robot motion and unexpected speed variations reduce the efficiency and reliability of the system. Conventional collision detection methods, focusing primarily on immediate collision avoidance, lead to jarring movements and path deviations that make the robot’s behavior appear unnatural and unpredictable, ultimately undermining trust in the autonomous navigation system.

To address this limitation, we propose a time-interval-based collision detection method integrating Light Detection and Ranging (LiDAR)-based sensing [24,25,26] with kinematic prediction. Similar to how insects use their antennae to sense and navigate their environment before physical contact, our system proactively predicts potential collisions by extending its perception into the near future. This predictive approach enables the robot to anticipate and respond to potential obstacles well before physical proximity triggers reactive behaviors.

This anticipatory approach combines three key elements for effective collision prediction. First, a kinematic model computes the robot’s future positions based on current state and control inputs. Second, LiDAR sensors provide high-resolution spatial data for human detection [27,28]. Third, Kalman filter-based estimation enables the accurate prediction of human trajectories [29], essential for proactive collision avoidance. The main contributions of this research are as follows:

1. Development of a time-interval-based collision detection system that integrates robot kinematics with human trajectory prediction, enabling proactive obstacle avoidance;
2. Implementation of a stable human tracking method utilizing 4WIS Parallel mode capabilities, which maintains consistent sensor orientation during navigation;
3. Experimental validation of the system’s effectiveness in realistic indoor scenarios, demonstrating improved navigation efficiency and reliability.

The remainder of this paper is organized as follows. Section 2 reviews related works in autonomous delivery robots, 4WIS systems, human detection, and collision avoidance. Section 3 presents the system architecture and methodologies. Section 4 demonstrates the experimental results validating our approach. Finally, Section 5 concludes with discussions on system effectiveness and future improvements.

## 2. Related Works

### 2.1. Indoor Delivery Robot Systems

Research in autonomous indoor delivery systems has focused on optimizing navigation and interaction capabilities in human-shared environments [7,8]. While various robotic platforms have been developed, ground-based mobile robots have emerged as the preferred solution for indoor environments [11,12,30], offering advantages in carrying capacity and operational stability. However, traditional mobile robot platforms often struggle with the complex requirements of indoor navigation, particularly in dynamic human-shared spaces.

### 2.2. 4WIS Mobile Robot Systems

4WIS systems represent a significant advancement in mobile robot technology [13,14,15], offering enhanced maneuverability and control precision critical for indoor operations. Researchers have made significant progress in improving the stability and performance of these systems through various control strategies. The integration of Direct Yaw-moment Control (DYC) with Active Steering (AS) has significantly improved vehicle lateral dynamics [31,32], while its combination with Model Predictive Control (MPC) has optimized system performance by reducing the impact of DYC on the longitudinal velocity [33]. Recent research has established four primary steering strategies for 4WIS robots as follows [17,18]:

- Ackermann steering for traditional car-like navigation [19,20,21];
- Parallel steering enabling lateral movement while maintaining orientation [22,23];
- Crab steering for combined forward and lateral motion [34];
- Spinning mode for in-place rotation [16].

Particularly significant for indoor applications is the parallel steering mode, which ensures consistent sensor orientation during movement [22,23]. This stability is crucial for maintaining reliable human detection and tracking, addressing a key limitation of conventional steering systems in dynamic indoor environments.

### 2.3. Human Detection and Tracking

LiDAR-based human detection has become fundamental for autonomous systems in human-shared environments [24,25,26], offering superior performance across various lighting conditions [35,36,37]. Recent advances in point cloud processing have significantly improved detection accuracy and reliability [38,39]. The integration of Kalman filtering for trajectory prediction [29,40,41] has enhanced real-time human tracking capabilities [42,43,44]. Recent research has focused on developing more sophisticated motion models [45], though challenges remain in predicting human movement patterns in unstructured indoor environments.

### 2.4. Collision Avoidance in Indoor Environments

Traditional collision avoidance approaches typically rely on reactive strategies based on immediate proximity detection [46,47,48]. While these methods ensure basic safety through threshold-based responses [49,50], they often result in inefficient robot behavior and reduced system reliability in shared spaces. Recent research has explored predictive approaches [51,52], attempting to anticipate potential collisions by considering future trajectories [53,54,55]. However, integrating human trajectory prediction with robot path planning remains challenging [56,57], particularly in dynamic indoor environments where human behavior can be unpredictable. While advanced mapping technologies [58,59] and motion planning techniques [60,61] have improved navigation capabilities, balancing efficient operation with system reliability remains an active research challenge [62,63,64]. Conventional methods particularly struggle with multiple moving obstacles [65,66], highlighting the need for more sophisticated approaches to collision prediction and avoidance [46,47,48].

These existing approaches demonstrate the need for an integrated solution that combines stable sensing capabilities with predictive collision detection for effective human–robot interaction in indoor environments.

## 3. Materials and Methods

### 3.1. Modelings of 4WIS Mobile Robot

**Figure 1:**
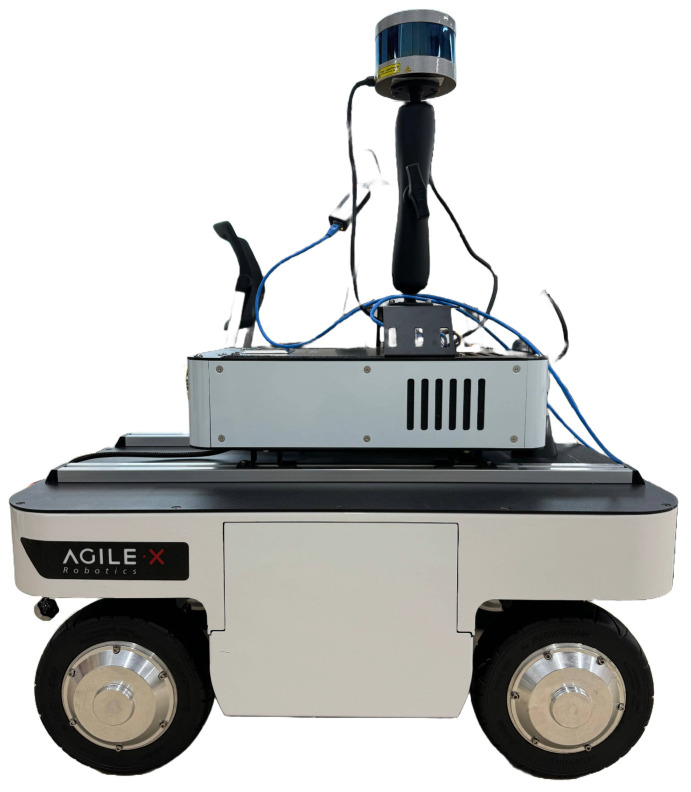
The 4WIS mobile robot platform used in this study.

Figure 1 shows the mobile robot used in this study. The 4WIS mobile robot was designed with specific characteristics optimized for indoor navigation. A key characteristic of the 4WIS system is that each wheel features independent steering and drive capabilities through individual in-wheel motors. The independent control of each wheel enables multiple driving modes, including conventional Ackermann steering for efficient straight-line motion and omnidirectional movement for complex maneuvers, allowing the robot to adapt to various navigation scenarios encountered in indoor environments.

### 3.2. Hardware Architecture

**Figure 2:**
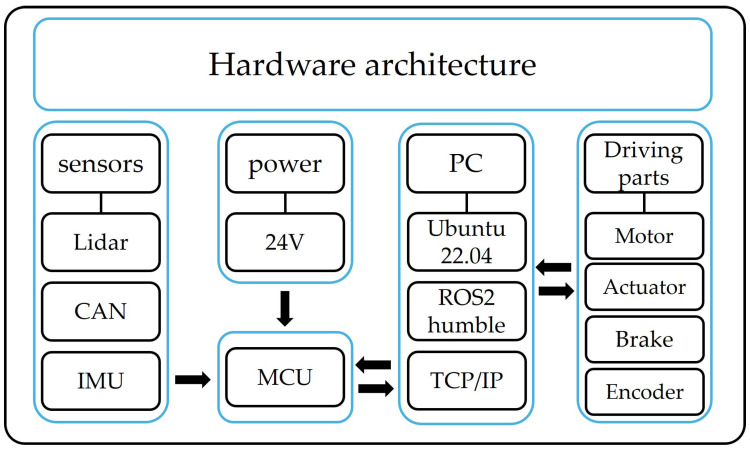
Hardware architecture of mobile robot.

Figure 2 shows the hardware architecture of the mobile robot used in this experiment. The robot, powered by a 24 V battery, is equipped with a LiDAR, camera, CAN BUS, and IMU as the primary sensors, which are connected to the MCU of the robot. The local PC facilitates sensor fusion between the driving components and MCU, acting as a data intermediary for various sensors. These data are utilized by the ROS2 internal algorithms to actuate the actuators and send feedback data back and forth.

### 3.3. Driving Mode of 4WIS Mobile Robot

**Figure 3:**
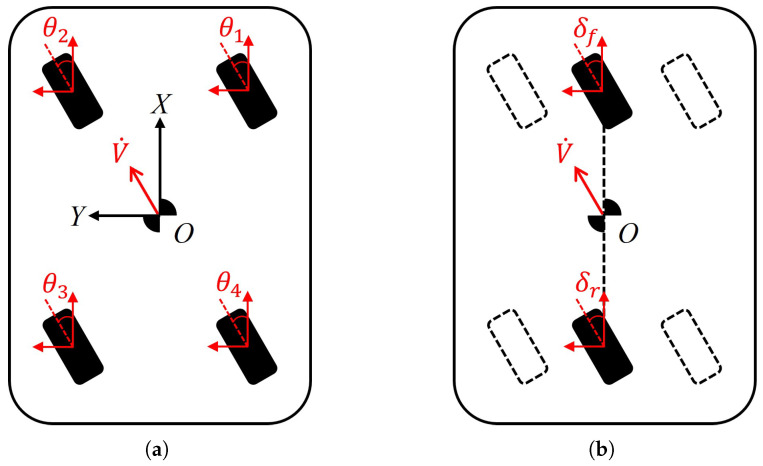
One of the primary driving methods employed by 4WIS mobile robot. (**a**) Illustration of the Parallel mode; (**b**) illustration of the bicycle model for Parallel mode.

Figure 3a illustrates the parallel driving mode, one of the key steering strategies of 4WIS mobile robots. In Parallel mode, there is no rotational movement, which distinguishes it from other modes like Ackermann steering. Instead, only longitudinal (X-direction of the robot) and transverse (Y-direction of the robot) movements are involved, allowing for precise control in two dimensions. The most notable feature of this mode is that all four wheels steer in the same direction simultaneously. Given that the front and rear wheels are situated on the same horizontal plane, the motion vector of the mobile robot in Parallel mode can be derived through the application of the bicycle model [67]:

(1) $$\dot{V}=\left[\begin{array}{c} V_{X}\\ V_{Y}\\ 0 \end{array}\right]$$

The bicycle model is a simplified representation commonly used in vehicle dynamics and control. In the context of 4WIS mobile robots operating in Parallel mode, this model is particularly useful due to the uniform steering of all wheels. As shown in Figure 3b, the bicycle model reduces the four-wheel system to a two-wheel equivalent, with one wheel representing the front axle and another representing the rear axle. In Parallel mode, all four independent wheels are steered at the same angle. Consequently, the front and rear wheels of the bicycle model in Parallel mode are also steered at the same angle:

(2) $$\delta_{f}=\delta_{r}$$

In this case, the steering angles of the wheels can be determined as follows:

(3) $$\theta_{1}=\theta_{2}=\theta_{3}=\theta_{4}=\tan^{-1}\left(\frac{V_{Y}}{V_{X}}\right)$$

This simplified model allows for easier analysis and control of the robot’s motion while still capturing its essential kinematic behavior.

### 3.4. Kinematics of Parallel Mode

**Figure 4:**
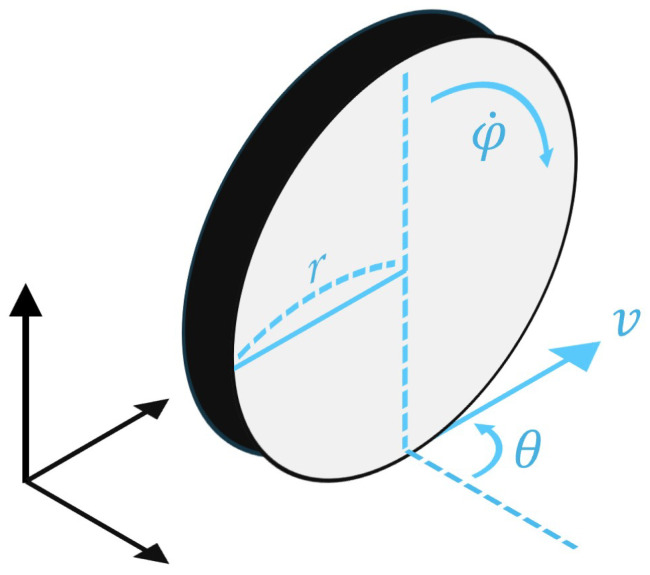
Wheel model in 3D coordinate system.

To develop the kinematic-based future trajectory prediction algorithm, we now introduce the process of obtaining a kinematic model for Parallel mode. This model is crucial for understanding and predicting the motion of the 4WIS mobile robot in indoor environments. When considering a wheel in a three-dimensional coordinate system, as shown in Figure 4, several key parameters define its motion. These parameters include the following:

- *r*: the radius of the wheel;
- $\dot{\phi }$: the speed of the wheel;
- θ: the direction of the wheel;
- *v*: the velocity of the wheel;
- ω: the rotational speed of the wheel.

(4) $$\left[\begin{array}{c} \dot{x}\\ \dot{y}\\ \dot{\theta } \end{array}\right]=\left[\begin{array}{cc} r\cos(\theta ) & 0\\ r\sin(\theta ) & 0\\ 0 & 1 \end{array}\right]\left[\begin{array}{c} \dot{\phi }\\ \omega \end{array}\right]$$

Building upon the single-wheel kinematic model, we can extend our analysis to the entire 4WIS mobile robot in Parallel mode [68,69]. In this instance, the expression for the velocity of each wheel (*v*) is approximately equal to $r\dot{\phi }$, which allows us to generalize it as follows:

(5) $$\left[\begin{array}{c} \dot{x}\\ \dot{y}\\ \dot{\theta } \end{array}\right]=\left[\begin{array}{cc} \cos(\theta ) & 0\\ \sin(\theta ) & 0\\ 0 & 1 \end{array}\right]\left[\begin{array}{c} v\\ \omega \end{array}\right]$$

This approximation simplifies our model while maintaining its accuracy for the purposes of trajectory prediction.

In accordance with Equation (5), we can express *s*, which represents the velocity of the mobile robot, as follows:

(6) s=vcos(θ)

This equation describes the overall motion of the 4WIS mobile robot in Parallel mode, taking into account the contributions of all four wheels. By utilizing these equations, we can effectively model and predict the motion of the 4WIS mobile robot in Parallel mode. This kinematic model serves as the foundation for our trajectory prediction algorithm, enabling us to accurately anticipate the robot’s movement in various indoor environments.

**Figure 5:**
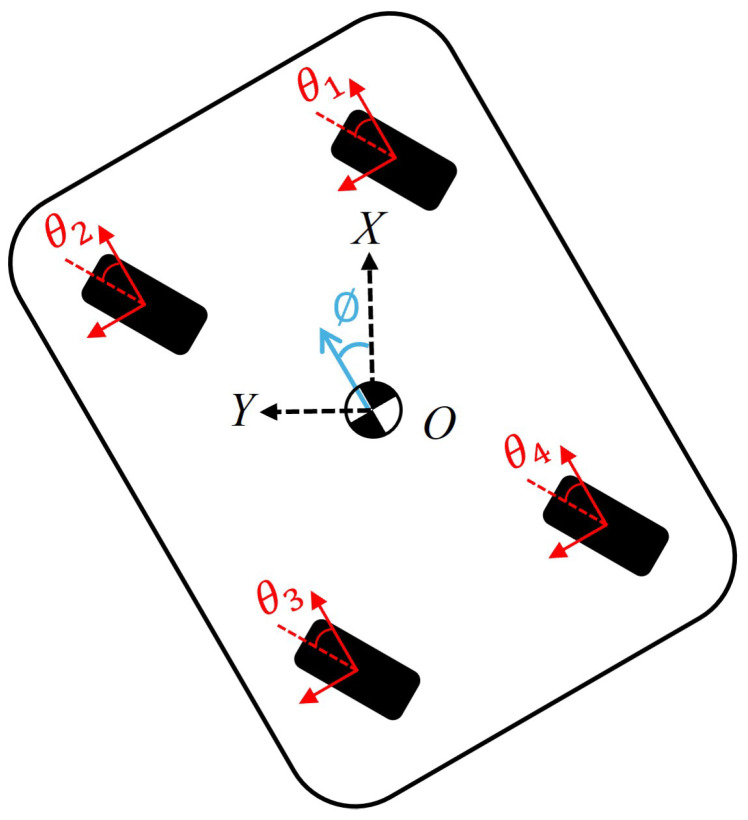
Kinematic model of Parallel mode.

Building upon our earlier equations, we can now develop a comprehensive kinematic model for the 4WIS mobile robot operating in Parallel mode. Figure 5 illustrates a representation of the Parallel mode drive, where θ represents the angle of rotation on the *X*-*Y* coordinate plane. This visual representation helps us understand how the robot’s orientation relates to its movement in two-dimensional space. The kinematic model of the Parallel mode can be represented as follows:

(7) $$\left[\begin{array}{c} \dot{x}\\ \dot{y}\\ \dot{\theta } \end{array}\right]=\left[\begin{array}{cc} \cos(\emptyset +\theta ) & 0\\ \sin(\emptyset +\theta ) & 0\\ 0 & 1 \end{array}\right]\left[\begin{array}{c} v\\ \omega \end{array}\right]$$

This model encapsulates the unique motion characteristics of the 4WIS robot in Parallel mode, allowing for precise control and trajectory prediction. To fully appreciate the advantages of this model, it is instructive to compare it with the kinematic model of the more traditional Ackermann steering system. Therefore, we briefly introduce the kinematic model for Ackermann mode used in this paper.

Under normal circumstances, vehicles typically operate in the Ackermann mode, which mimics the driving style of a car. The Ackermann mode is a crucial component in modern automobiles as well as in autonomous and robotic vehicles.

**Figure 6:**
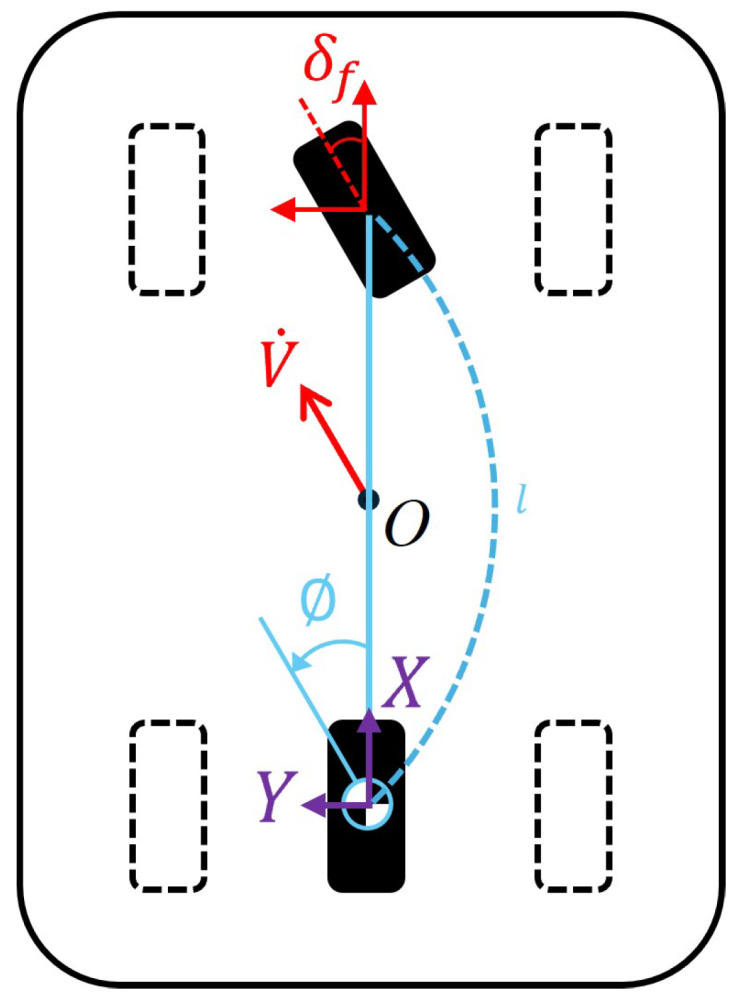
Kinematic model of Ackermann mode.

Figure 6 illustrates the kinematic model for the Ackermann mode. The kinematic model of the Ackermann mode is expressed as follows:

(8) $$\left[\begin{array}{c} \dot{x}\\ \dot{y}\\ \dot{\theta } \end{array}\right]=\left[\begin{array}{cc} \cos(\emptyset ) & 0\\ \sin(\emptyset ) & 0\\ \tan(\delta_{f}/l) & 1 \end{array}\right]\left[\begin{array}{c} s\\ \omega \end{array}\right]$$

### 3.5. Control System Architecture

**Figure 7:**
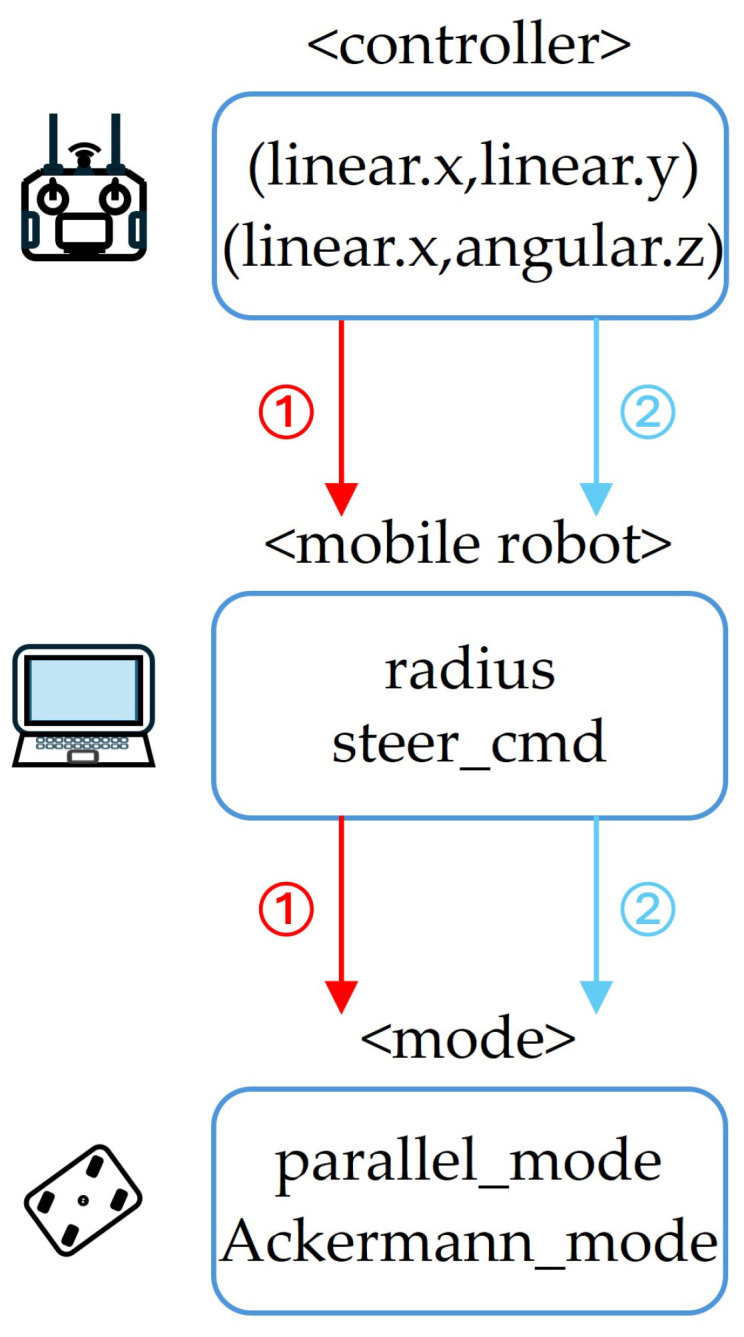
Flowchart for controlling a mobile robot.

Figure 7 shows a flowchart of the control system for the mobile robot. The mobile robot accepts two command formats for velocity control: (linear.x, linear.y) and (linear.x, angular.z). The ROS2 internal algorithm on a local PC receives these data and is responsible for interpreting the command format, calculating the turning radius or steering angle, and switching the mobile robot to the appropriate driving mode. Previously, the driving mode was preset based on the signal format: if the signal included lateral movement (linear.y), the robot operated in Parallel mode; if it included rotational movement (angular.z), it switched to Ackermann driving mode.

### 3.6. Mobile Robot Trajectory Calculation

Our method for determining the appropriate steering angles and rotation directions is based on the received command signals. We use a simple yet effective criteria system to translate commands into wheel configurations.

**Figure 8:**
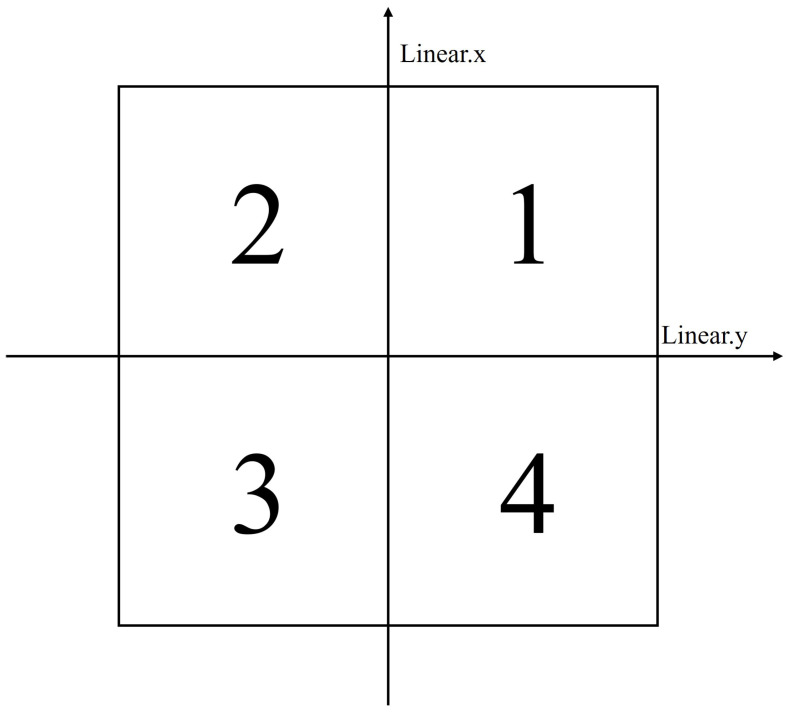
Quadrant-based steering angle determination for wheel control.

Figure 8 and Table 1 illustrate how we determine the steering angle and wheel rotation direction based on the control signals and the quadrant in which the wheel is located. This system’s simplicity allows for rapid computation, enabling real-time adjustments even in fast-changing environments.

For example, if Linear.x > 0 and Linear.y > 0, the wheel is located in the first quadrant (1/4 quadrant). In this case, according to Table 1, the steering angle is positive (+), and the wheel direction is front. This means the wheel will be oriented towards the positive direction of the x-axis and y-axis and will rotate forward. Here, we can find that the direction of the wheel is determined by Linear.x and the steering angle is determined by Linear.y. Based on this relationship, we can rewrite Equation (3) using our control signal format as follows:

(9) $$\theta_{1}=\theta_{2}=\theta_{3}=\theta_{4}=q\times \left|\tan^{-1}\left(\frac{V_{Y}}{V_{X}}\right)\right|$$

where *q* represents the sign of steering angle from Table 1, and $V_{X}$, $V_{Y}$ are the velocity components corresponding to Linear.x and Linear.y, respectively.

This approach offers a significant advantage: it potentially decreases the time required for wheel adjustments during direction changes. This improvement in responsiveness can be crucial in dynamic indoor environments where quick adaptations are often necessary. Moreover, the reduced adjustment time can lead to smoother overall motion and potentially lower energy consumption.

Having established our efficient method for determining wheel configurations, we now turn our attention to predicting the robot’s trajectory over time. While our innovative approach to steering angle and rotation direction determination provides precise and immediate control over the robot’s movement, we must further develop this into a comprehensive system for collision avoidance. In the following sections, we will detail our kinematic model and demonstrate its effectiveness in predicting robot trajectories and avoiding collisions in various indoor scenarios.

To begin this analysis, we present a kinematic approach to predict and visualize the future position of a mobile robot. This approach facilitates the detection of collisions between the robot and people. Kinematics is a mathematical approach to describing the motion of a robot, enabling the representation of state variables such as position and velocity as a function of time. In this study, we assume motion in a two-dimensional plane and model the robot’s position using the following equations:

(10) $$x\left(t\right)=x_{0}+v_{x}\times t$$

(11) $$y\left(t\right)=y_{0}+v_{y}\times t$$

where x(t) and y(t) are the position of the robot after time *t*, $x_{0}$ and $y_{0}$ are the current position, and $v_{x}$ and $v_{y}$ are the velocities along the *x* and *y* axes, respectively.

Building upon these kinematic principles, we can determine the values of $v_{x}$ and $v_{y}$ for our specific robot configuration. These values are calculated as follows:

(12) $$v_{x}=s\cos\left(\theta \right)$$

(13) $$v_{y}=s\sin\left(\theta \right)$$

By applying these equations, we can accurately estimate the robot’s future position by comparing the predicted values with the actual movement values obtained through the robot’s internal encoder:

(14) $$x\left(t\right)=x_{0}+s\cos\left(q\times \left|\tan^{-1}\left(\frac{V_{Y}}{V_{X}}\right)\right|\right)\times t$$

(15) $$y\left(t\right)=y_{0}+s\sin\left(q\times \left|\tan^{-1}\left(\frac{V_{Y}}{V_{X}}\right)\right|\right)\times t$$

A key feature of our kinematic model is the inclusion of the time parameter t. This parameter provides our system with the flexibility to predict the robot’s position at various future time points. By adjusting the value of t, we can anticipate potential collisions not just in the immediate future but at multiple time intervals ahead. This capability is particularly crucial during path tracking, where the robot must dynamically respond to potential collisions while following a pre-generated path, ensuring safe human–robot interaction in indoor environments.

**Figure 9:**
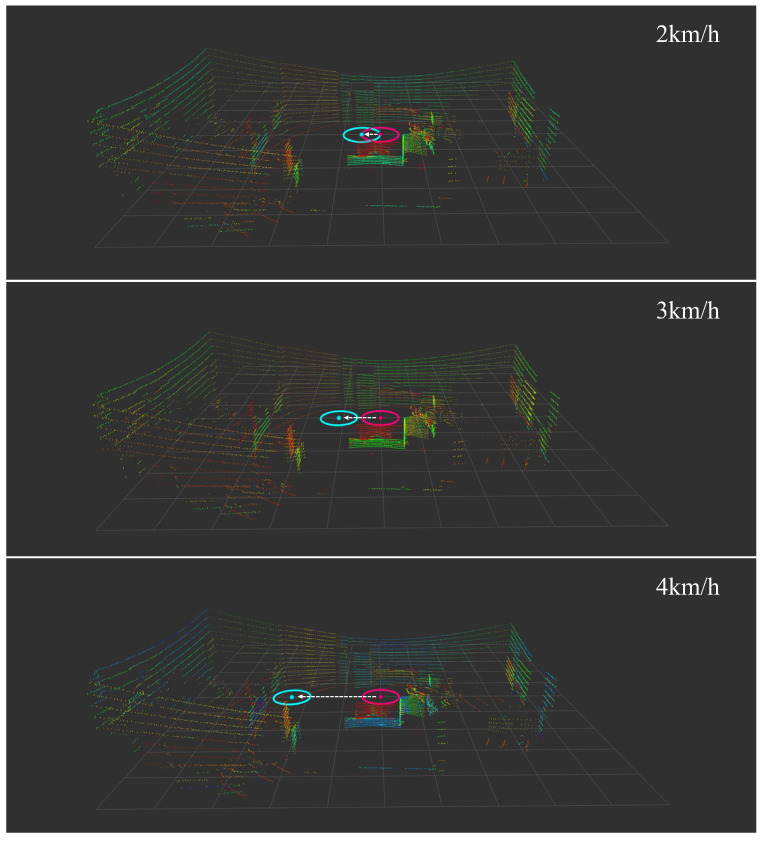
Example of mobile robot trajectory calculation 3 s later in different speed setting.

As illustrated in Figure 9, our trajectory calculation algorithm visually distinguishes between the current and predicted positions of the mobile robot. The current location of the robot is represented by a red circle, while the predicted future location is shown as a blue circle. This intuitive visualization allows users to easily understand the anticipated movement of the robot. In the figure, each grid cell represents an area of 100 cm by 100 cm, providing a clear scale for the robot’s movement.

**Figure 10:**
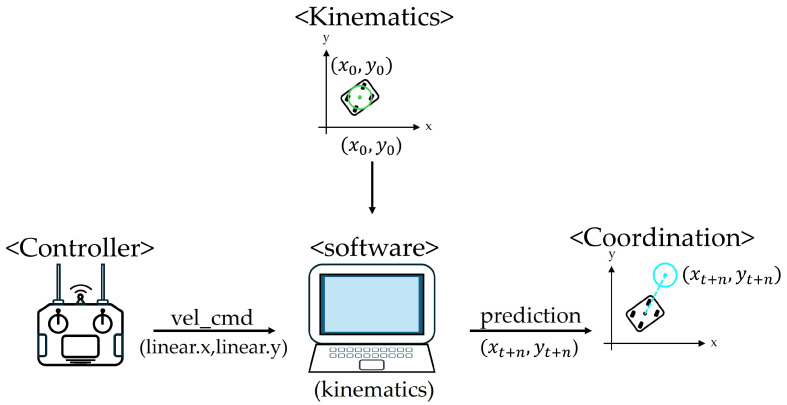
Overall structure of mobile robot trajectory calculation.

Figure 10 illustrates the overall structure of mobile robot trajectory prediction. The algorithm receives the velocity command sent from the controller in the form of (Linear.x, Linear.y) and uses the kinematic equations of the mobile robot to return the coordinates of the expected positions in a few seconds. It calculates this from the current position ($x_{0}$, $y_{0}$) to the predicted center position of the robot.

To conclude, this section presented a steering control strategy using kinematics and steering rules, along with a trajectory calculation algorithm that can predict the mobile robot’s position at desired time intervals. The proposed method effectively reduces the response time for path adjustments while maintaining precise control through our quadrant-based steering system.

### 3.7. Human Detection

**Figure 11:**
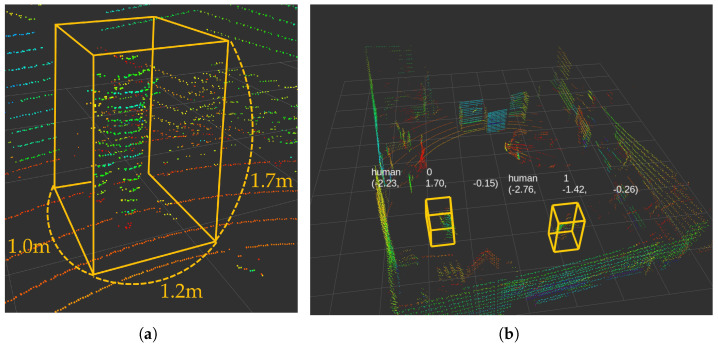
(**a**) Preset human size parameters; (**b**) real-time human detection results.

The LiDAR-based human detection system, demonstrated in Figure 11, operates within a 5 m radius from the robot’s center, providing optimal coverage for indoor environments. This specific distance is chosen as an optimal balance between safety and operational efficiency. It takes into account typical human walking speeds, the mobile robot’s maximum speed, and provides sufficient time for the robot to react and adjust its trajectory if needed.

To enhance the accuracy of the recognition process, we predefine the range of human size parameters to filter potential human candidates [38,39] As shown in Figure 11a, these preset values for human detection are width 0.5∼1.0 m, length 1.0∼1.2 m, and height 1.5∼1.7 m. The point cloud data obtained from the LiDAR sensor are then clustered based on these parameters to detect people.

**Figure 12:**
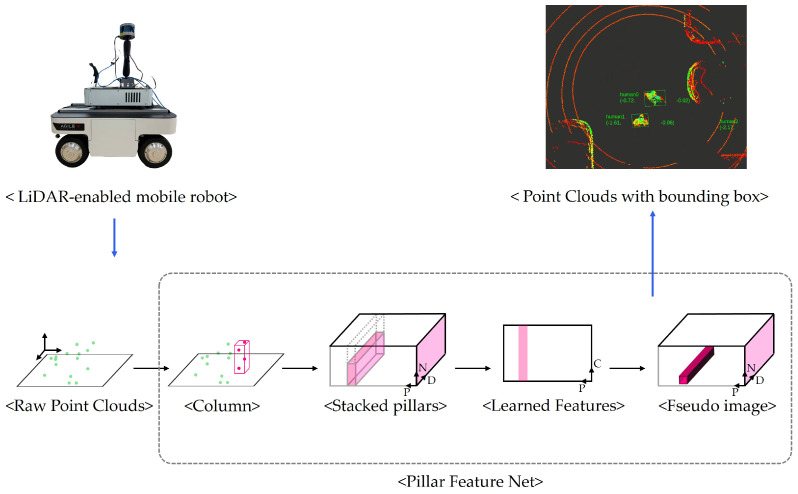
Human detection algorithm architecture.

For detecting human objects (LiDAR-based 3D object) in point cloud data, we employ the Pillar Feature Net algorithm, as shown in Figure 12, which transforms irregular 3D LiDAR points into an organized grid structure through vertical discretization (pillars) [70,71]. The algorithm encodes point-wise features and spatial relationships within each pillar, converting the point cloud data into a pseudo-image format that can be efficiently processed by conventional 2D convolutional networks for real-time human detection.

### 3.8. Human Trajectory Prediction

**Figure 13:**
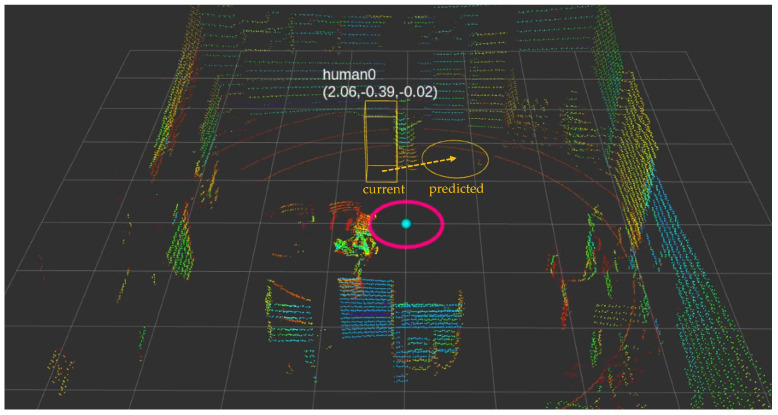
Visualization of real-time trajectory prediction results.

The integration of LiDAR detection with Kalman filter-based prediction [29,40,41] enables robust human trajectory estimation. Figure 13 demonstrates our system’s ability to track and predict human movements in real-time, with yellow rectangles indicating current positions and yellow circles showing predicted future locations [42,43]. This prediction framework, combined with our Parallel mode’s stable sensor orientation [63], enables more reliable human tracking compared to conventional approaches [28,45].

The Kalman filter operates through an iterative two-step process:

- Prediction step: estimates future states based on the current state and system model;
- Update step: refines these predictions using actual measurements.

This continuous cycle of prediction and correction allows our system to accurately track and predict pedestrian movements. By combining robust human detection with the Kalman filter’s predictive capabilities, our system provides reliable trajectory predictions for pedestrians in the robot’s vicinity, enabling safer human–robot interaction in indoor environments. A demonstration of the proposed method in real-world scenarios is provided in Supplementary Video S1, available in the Supplementary Materials Section.

In this study, we implement a linear Kalman filter to track and predict the positions of humans detected in a point cloud environment [43,44]. The formulation and application of the Kalman filter in our method can be summarized as follows:

(16) $$x=\left[\begin{array}{c} x_{pos}\\ y_{pos}\\ x_{vel}\\ y_{vel} \end{array}\right]$$

This state vector encapsulates both position and velocity information of the detected objects.

The state transition matrix A is defined as

(17) $$A=\left[\begin{array}{cccc} 1 & 0 & 1 & 0\\ 0 & 1 & 0 & 1\\ 0 & 0 & 1 & 0\\ 0 & 0 & 0 & 1 \end{array}\right]$$

This model predicts the currents state based on the previous state, incorporating changes in position due to velocity.

The measurement model is defined as

(18) $$H=\left[\begin{array}{cccc} 1 & 0 & 0 & 0\\ 0 & 1 & 0 & 0 \end{array}\right]$$

However, in our system, the matrix only measures position information, neglecting velocity data. This is because simultaneously and continuously tracking both position and velocity for each person is challenging in real-world scenarios. Therefore, we propose an alternative approach which is detailed in this section.

In the prediction step, the prediction state and covariance equation of the algorithm at time (t−1) are

(19) $$\hat{x}=A{\hat{x}}_{k-1}+B\mu_{k}$$

(20) $$P=AP_{k-1}A^{T}+Q$$

The control input matrix typically represents the effect of external control inputs on the state. In many scenarios, such as the one presented here, this could include forces or acceleration acting on the detected object. However, in the current implementation, *B* is not explicitly utilized, as no control inputs are defined or applied.

In the update step, the Kalman gain *K* is calculated using Equation (21), where *R* represents the measurement noise covariance matrix. The matrix *R*, defined in Equation (22), reflects the uncertainty in our measurements. When the sensor measurements are highly accurate, *R* values can be set lower, while less accurate measurements require higher *R* values. This allows our system to be adaptable to different sensor characteristics and environmental conditions:

(21) $$K=PH^{T}{\left(HPH^{T}+R\right)}^{-1}$$

(22) $$R=\left[\begin{array}{cc} 1 & 0\\ 0 & 1 \end{array}\right]$$

According to these equations, the predicted state can be updated using the measurement vector z, which contains the observed position coordinates:

(23) $$\hat{x}=\hat{x}+K\left(z-H\hat{x}\right)$$

(24) $$z=\left[\begin{array}{c} z_{x}\\ z_{y} \end{array}\right]$$

where $z_{x}$ and $z_{y}$ represent the measured positions in the *x* and *y* directions, respectively. These measurements are crucial for updating the state estimates in the Kalman filter, allowing us to correct our predictions based on actual observations.

**Figure 14:**
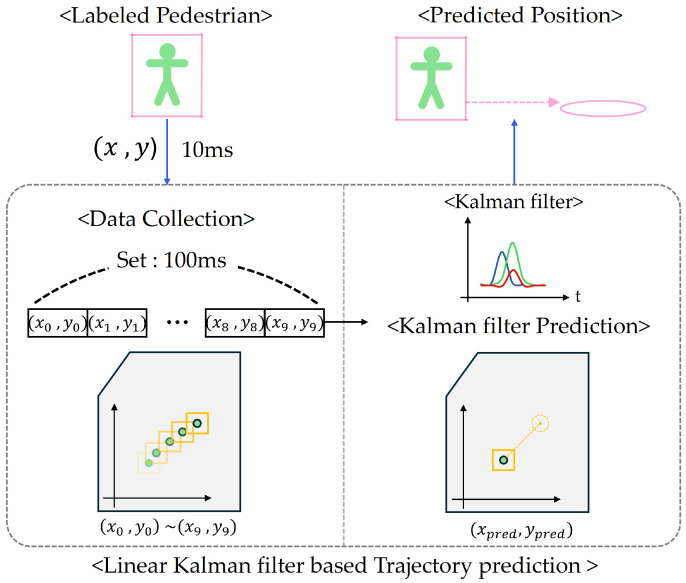
Trajectory prediction system architecture.

Figure 14 illustrates the overall structure of our trajectory prediction system. To address the limitations of velocity measurements, our system collects 10 consecutive position observations at 10 ms intervals over a 100 ms period. The algorithm processes these position data through a state transition model and observation updates, continuously improving prediction accuracy through iterative refinement. This approach simplifies the tracking process while maintaining prediction accuracy by focusing solely on position data, which has proven effective in dynamic indoor environments where continuous velocity tracking is challenging.

### 3.9. Time Interval Collision Detection

In this section, we present our proposed collision detection algorithm that combines kinematic-based trajectory calculation and Kalman filter-based human trajectory prediction. Traditional collision detection approaches typically follow a sequential process: first generating a path on a map [46,47,48], then having the mobile robot navigate along this path while continuously correcting its position through localization [51,52]. During this process, motion planning enables the mobile robot to predict its position along the generated path at different time intervals.

**Figure 15:**
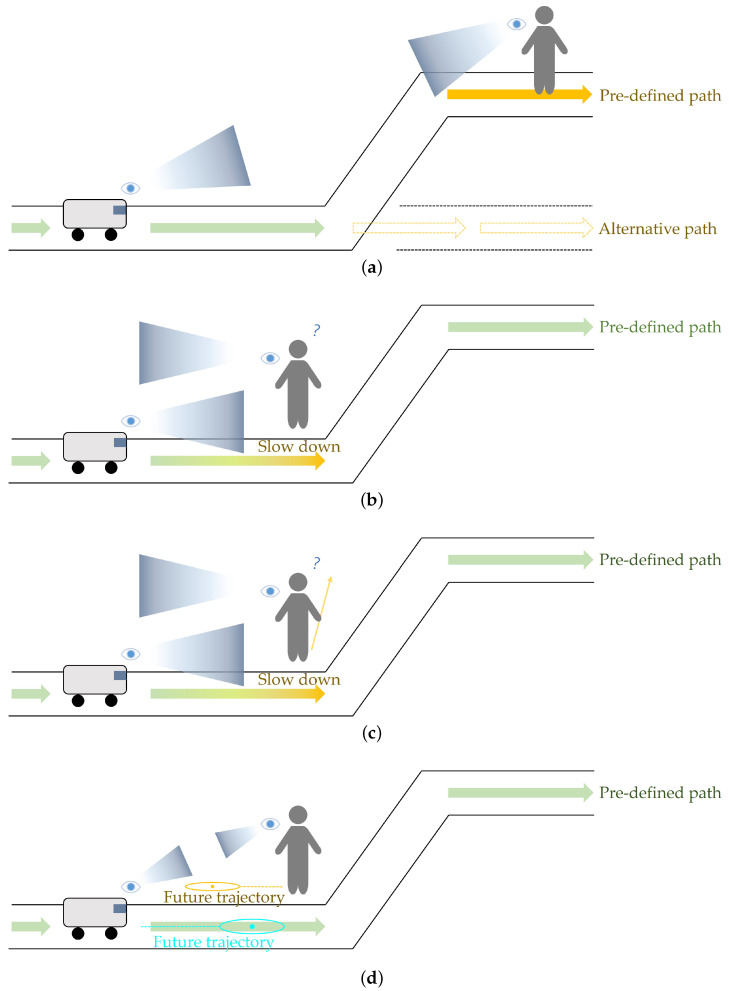
Collision detection approaches: (**a**) Traditional path-based detection; (**b**) Conventional method limitations; (**c**) Unnecessary collision responses; (**d**) Proposed trajectory prediction method.

Collision detection in mobile robotics can be categorized into two distinct scenarios. The first scenario occurs when an object is detected directly on the robot’s generated path [54,55]. In this case, traditional approaches either generate a new collision-free path or temporarily halt the robot until the moving object passes. This approach is relatively intuitive for humans sharing the space, as they can easily recognize that the robot is aware of their presence through its proactive responses and predict its next movements as shown in Figure 15a.

However, the second scenario, where potential collisions occur outside the generated path, has been handled less effectively by conventional methods [65,66] as illustrated in Figure 15b. Traditional approaches simply rely on LiDAR sensor’s detection radius, reducing speed or stopping whenever an object comes within a certain distance, regardless of its direction or movement pattern. This simplistic approach often results in unnecessary robot movements and stops, potentially causing discomfort and inconvenience to people sharing the same space, as the robot’s movements appear unnatural from a human perspective.

While traditional approaches may be appropriate when people are walking directly towards the robot’s planned path or the robot itself, they become problematic and unnatural when people are moving away from the path or the robot. In such cases, the robot’s reactive movements are unnecessary and can create awkward interactions as shown in Figure 15c. This not only leads to inefficient navigation but also undermines overall trust in the autonomous navigation system, as humans observe seemingly irrational responses to non-threatening situations.

To address these limitations in conventional path following, we propose mounting our prediction-based algorithm onto existing systems to enable smoother operation [46,47,48]. The collision detection system integrates the trajectory prediction algorithms of both the mobile robot and humans. Both algorithms return predicted positions in (x,y) coordinates, allowing comparison between the mobile robot’s anticipated path and the predicted trajectories of nearby pedestrians. A potential collision is identified when the predicted positions from both algorithms are within a 1-m radius of each other. This system allows for real-time collision risk assessment and proactive avoidance. The 1-m threshold is chosen based on the robot’s dimensions and typical human personal space. Figure 15d illustrates this collision detection algorithm.

**Figure 16:**
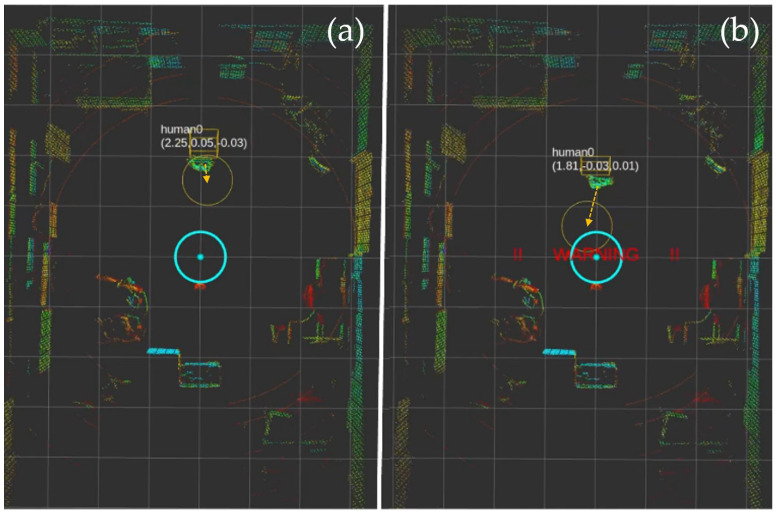
Collision detection system visualization.

Figure 16 shows the real-time visualization of our collision detection system in action. The sequence of three frames demonstrates how the system tracks and predicts human movement. The mobile robot’s position is indicated by the cyan cross at the center, while the detected human is represented by the yellow marker with coordinates displayed above. A cyan circle around the robot and a separate circle around the human indicate their respective detection and prediction zones. The visualization includes coordinate information (x,y) for precise position tracking, enabling the real-time assessment of potential collision risks [53,54,55]:

(25) $$Z=\{\left(x,y\right)|\sqrt{{\left(x_{r}-x_{h}\right)}^{2}+{\left(y_{r}-y_{h}\right)}^{2}}\leq d\}$$

where *Z* is the collision risk zone, $\left(x_{r},y_{r}\right)$ is the robot position, $\left(x_{h},y_{h}\right)$ is the human position, and *d* is the safety threshold distance (1 m).

### 3.10. System Integration and Workflow

**Figure 17:**
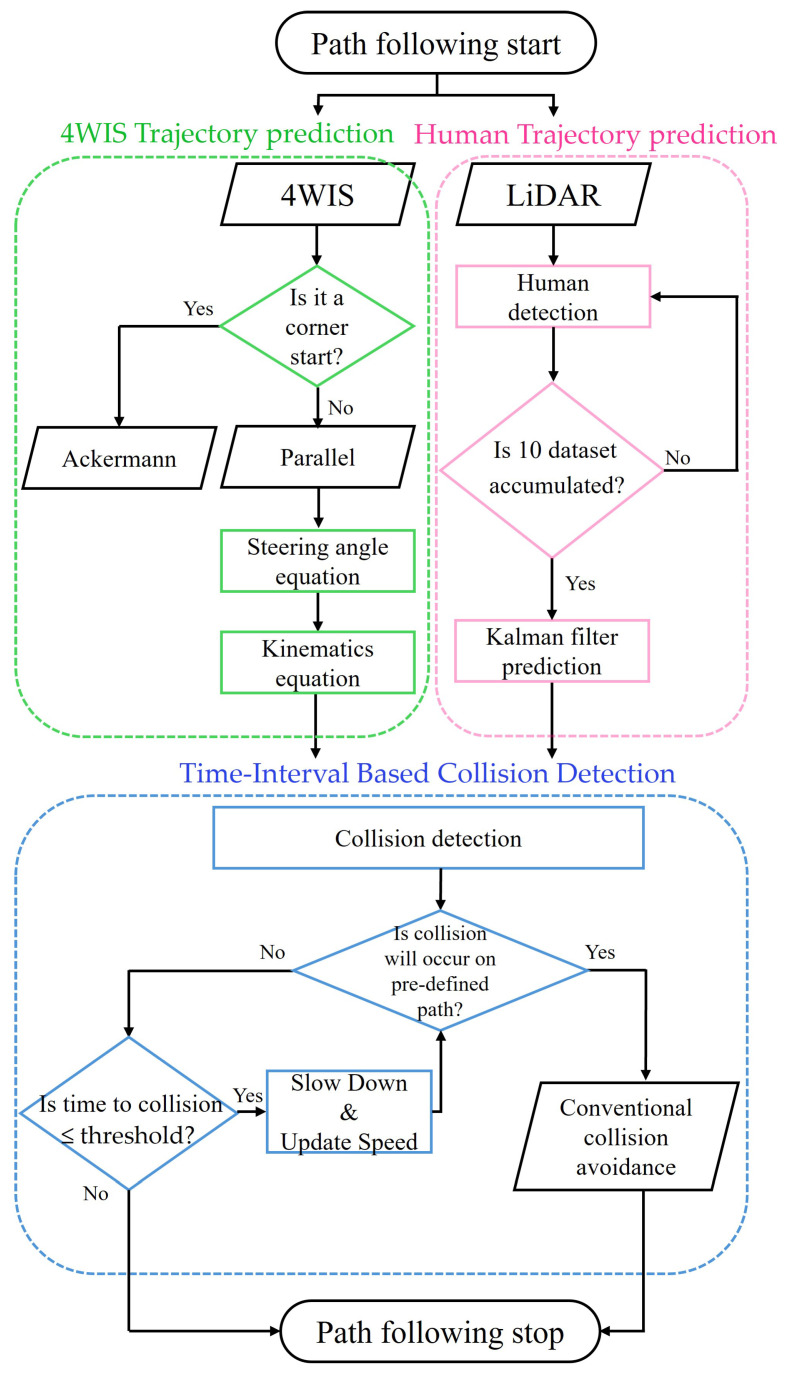
Overall flowchart of proposed collision detection system.

Figure 17 illustrates the complete workflow of our proposed system, which integrates path following, human detection, and collision prediction. The process consists of two parallel streams: robot control and human tracking. On the robot control side, the system determines the appropriate driving mode (Ackermann or Parallel) based on whether the robot is starting from a corner. In Parallel mode, the system utilizes steering angle and kinematic equations to calculate the robot’s trajectory.

Simultaneously, the LiDAR-based human detection system continuously monitors the environment. When a person is detected, the system accumulates 10 consecutive position measurements before initiating Kalman filter predictions. These two streams converge in the collision detection module, which compares the predicted trajectories of both the robot and detected humans.

The collision response operates in two distinct scenarios. When a collision is predicted on the predefined path, the system immediately activates conventional collision avoidance procedures. However, for potential collisions detected outside the path, the system employs a more nuanced approach, implementing a graduated speed reduction based on the predicted time-to-collision. Specifically, the robot reduces its speed to 80% when a collision is predicted 4 s ahead, further decreases to 60% at 3 s, drops to 30% at 2 s, and comes to a complete stop if a collision is predicted within 1 s. This graduated response system enables smooth and predictive collision avoidance while maintaining efficient path following operations. The effectiveness of this integrated system was validated through comprehensive experiments, which will be presented in the following section.

## 4. Results

### 4.1. Experiments Setup

**Figure 18:**
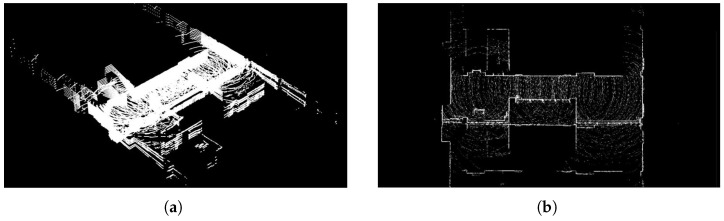
HD map of experimental environment. (**a**) A 3D point cloud visualization of the test environment; (**b**) Top-view representation of the indoor space showing the corridor and room layouts.

**Figure 19:**
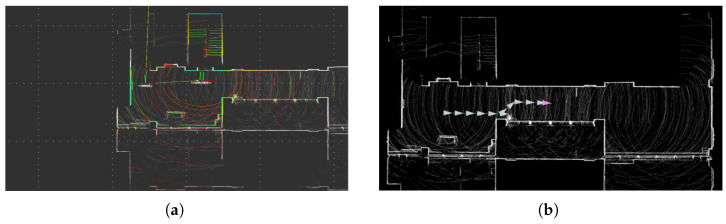
Environment mapping and localization system: (**a**) NDT matching visualization for real-time localization; (**b**) generated path planning overlay on the constructed HD map.

To validate our proposed collision detection and avoidance system, we conducted experiments in indoor environments by integrating our algorithm with an autonomous navigation system. We first created a detailed 3D vector map of the indoor environment using LiO-SAM (Tightly-coupled Lidar Inertial Odometry via Smoothing and Mapping) technology. This high-definition map served as the foundation for planning experimental paths that would test our collision avoidance capabilities [58,59]. The experimental environment, as shown in Figure 18, consists of corridors and rooms typical of indoor spaces where mobile robots might operate. Figure 18a presents the 3D point cloud visualization of our test environment, while Figure 18b provides a top-view representation that clearly shows the layout of corridors and rooms. Figure 19a shows the NDT matching process used for real-time robot localization, while Figure 19b illustrates the pre-planned path overlaid on the HD map, with white arrows indicating the robot’s intended movement direction through the space [25,60]. The specifications of the 4WIS mobile robot are shown in Table 2. The Experimental specifications and parameters are shown in Table 3.

The experiments were conducted on a 4.40 GHz Intel^®^ Core™ i5-1240P laptop with 16 GB of RAM, interfacing with Agilex’s ranger mini platform. This setup allowed us to process the sensor data and run our algorithms in real-time while controlling the mobile robot.

**Figure 20:**
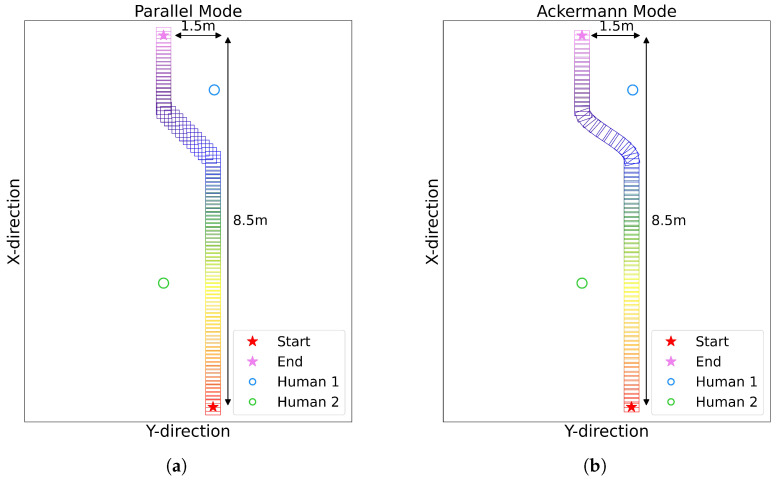
First experimental setup and real-time visualization of the mobile robot path.

Figure 20 illustrates our experimental setup for comparing the Parallel and Ackermann modes. Figure 20a shows the experiment conducted in Parallel mode, while Figure 20b depicts the same path followed in Ackermann mode. In both cases, the mobile robot followed a preset path: moving 5.5 m straight forward, then turning left slightly before continuing forward. This path was designed to simulate typical situations that a mobile robot might encounter while navigating indoor environments [38,39,72].

**Figure 21:**
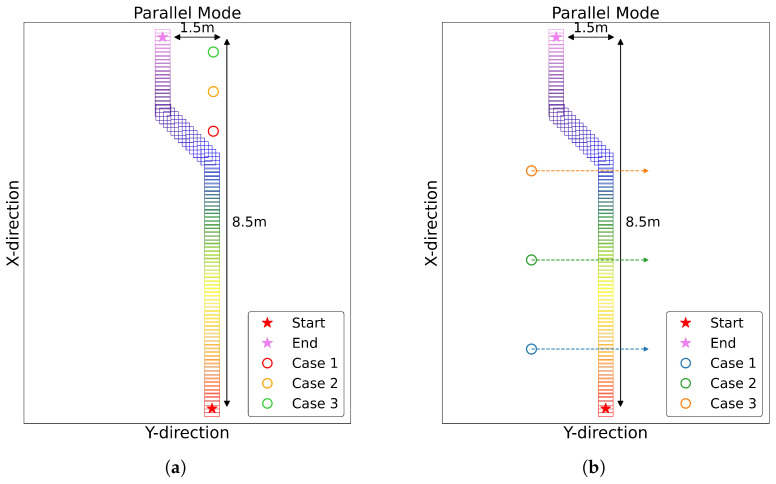
First experimental setup and real-time visualization of mobile robot path.

The second set of experiments was conducted to validate the performance of our proposed algorithm. Figure 21a,b demonstrate our testing setup for evaluating the time-to-collision (TTC) detection capabilities at varying distances, with each setup designed to test different aspects of the system’s performance. For both experimental setups, we conducted tests at two different mobile robot speeds: 2 km/h and 3 km/h.

In Figure 21a, we position the human subjects at three distances from where the robot’s avoidance movements start, to verify the different TTC results for different positions:

- Blue position: 0.5 m away;
- Green position: 1.75 m away;
- Orange position: 3.5 m away.

Figure 21b is designed to evaluate the time-to-collision (TTC) detection efficiency at varying distances, particularly focusing on how the robot performs in Parallel mode when human paths intersect with the robot’s trajectory. Human subjects walk 3 m while maintaining different initial distances from the robot’s Y-directional movement start point:

- Blue position: 1.45 m away;
- Green position: 3.25 m away;
- Orange position: 5.0 m away.

This setup allows us to evaluate not only the robot’s collision detection capabilities but also its potential for interaction with humans. The system distinguishes the possibility of collision over time, testing its performance with humans at various proximity to its path.

### 4.2. Experiments 1

**Figure 22:**
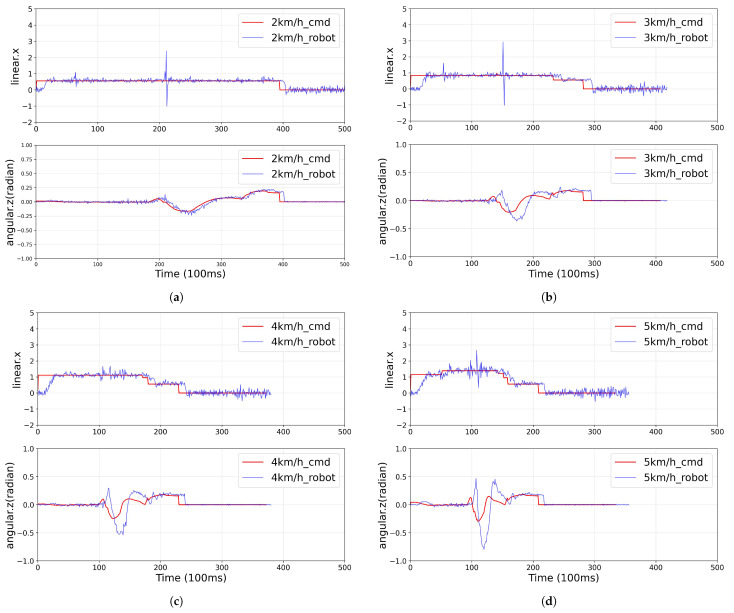
Comparison between command velocities and actual velocities of a 4WIS mobile robot in Ackermann mode during autonomous driving: (**a**) 2 km/h, (**b**) 3 km/h, (**c**) 4 km/h, and (**d**) 5 km/h. The red lines represent command values and blue lines show the actual robot response. The results demonstrate increasing trajectory tracking errors as the autonomous driving speed increases, particularly noticeable in both the linear velocity (linear.x) and angular velocity (angular.z) measurements.

The experimental results shown in Figure 22 demonstrate the velocity tracking performance of the 4WIS mobile robot operating in Ackermann mode at different speeds. The linear velocity (linear.x) and angular velocity (angular.z) data reveal a clear correlation between driving speed and control accuracy. At lower speeds (2 km/h and 3 km/h), the robot shows relatively stable tracking with minimal deviation from the command values. However, as the speed increases to 4 km/h and 5 km/h, there is a noticeable increase in the tracking errors, particularly during directional changes.

This degradation in tracking accuracy can be attributed to factors such as increased dynamic effects, mechanical limitations, and control system response times at higher speeds. The angular velocity plots specifically show larger oscillations and delayed responses at higher speeds, indicating challenges in maintaining precise steering control as velocity increases. These unstable motions and oscillations can significantly impact the performance of human detection systems, as the inconsistent sensor orientation during high-speed operations may lead to degraded detection reliability and reduced tracking accuracy. This finding emphasizes the importance of maintaining stable sensor orientation for reliable human detection and tracking in dynamic indoor environments.

**Figure 23:**
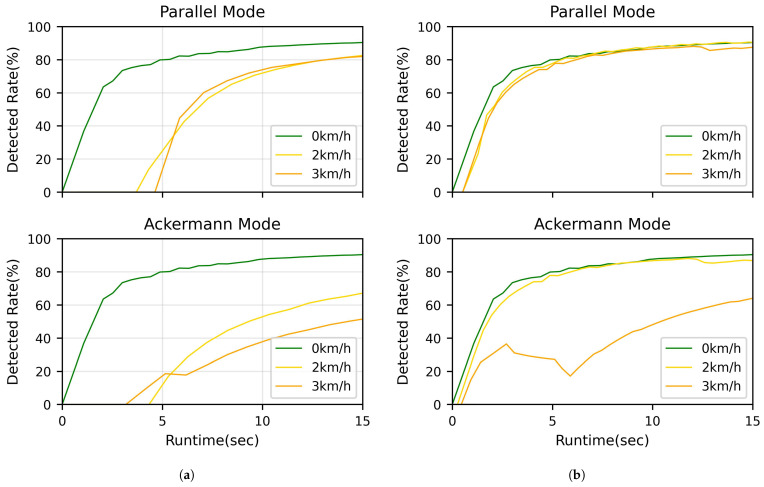
Human detection performance in Parallel and Ackermann modes at various speeds.

Figure 23a illustrates the detected rates of human object 1 over time for both Parallel and Ackermann modes at different speeds. In Parallel mode, the detection rates remain similar for both 2 km/h and 3 km/h speeds. However, in Ackermann mode, we observe a drop in detection rate as the speed increases, especially when the left turn starts. The detection rate is obtained by calculating the ratio of human detection time to elapsed time.

The 0 km/h speed serves as a control group, showing the detection rates when the mobile robot is stationary. The 2 km/h and 3 km/h speeds are chosen, as they represent the typical speeds used by mobile robots in indoor environments for safety reasons. This comparison reveals that Parallel mode maintains consistent detection performance across different speeds, while Ackermann mode’s performance degrades at higher speeds. This finding suggests that Parallel mode may be more suitable for maintaining reliable human detection in dynamic indoor environments.

Figure 23b presents the comparison results for human object detection with two people present. In Parallel mode (a), the mobile robot demonstrates stable detection of both human objects throughout its entire path. In contrast, Ackermann mode (b) exhibits difficulties in detecting human objects, particularly when executing a left turn. These results highlight the superior performance of Parallel mode in maintaining consistent human detection in indoor environments. The ability to reliably detect multiple human objects, even during turns, suggests that Parallel mode is better suited for navigation in dynamic indoor spaces where humans are present.

### 4.3. Experiments 2

**Figure 24:**
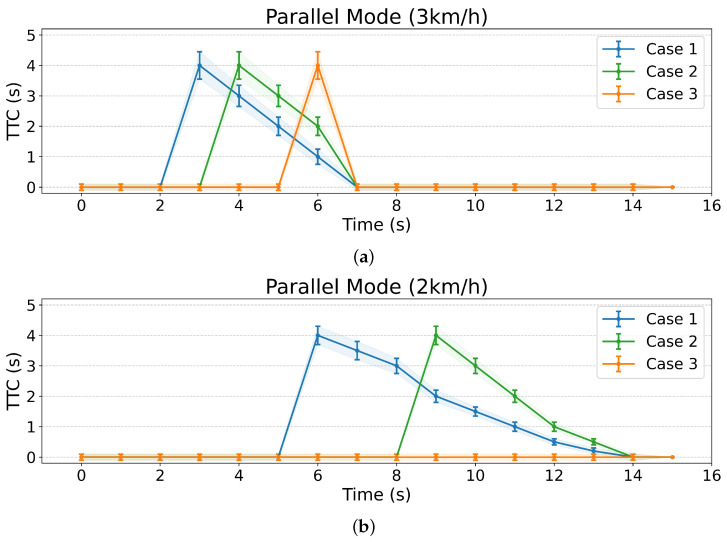
Time-to-collision prediction for multiple human positions in Parallel mode.

Figure 24 presents the experimental results of Figure 21. These findings demonstrate that our proposed algorithm can not only detect potential collisions with humans at different positions but also calculate the time remaining until a collision might occur. This capability is expected to enable more natural interactions with humans by allowing the robot to define its reactions based on the time-to-collision information.

**Figure 25:**
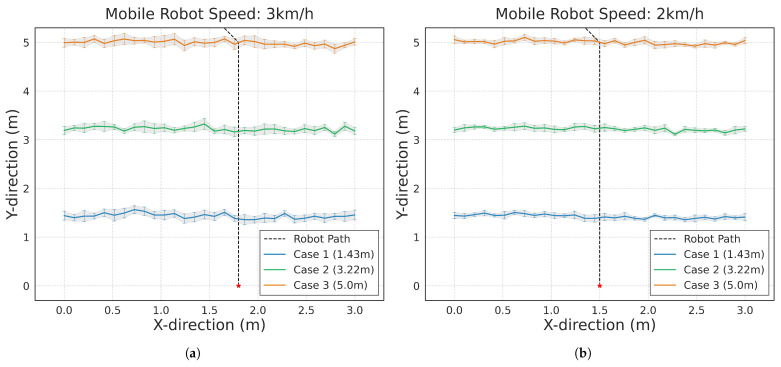
Human tracking results: (**a**) mobile robot speed of 2 km/h, (**b**) mobile robot speed of 3 km/h.

Figure 25 demonstrates the mobile robot’s human tracking performance as test subjects moved along a predetermined path covering 3 m, starting from three different initial positions. The experiments were conducted at two different robot speeds: 2 km/h (a) and 3 km/h (b). The results show successful tracking across all test cases, with the robot maintaining consistent tracking performance regardless of the humans’ starting positions at 1.43 m (Case 1), 3.22 m (Case 2), and 5.0 m (Case 3) from the start line.

**Figure 26:**
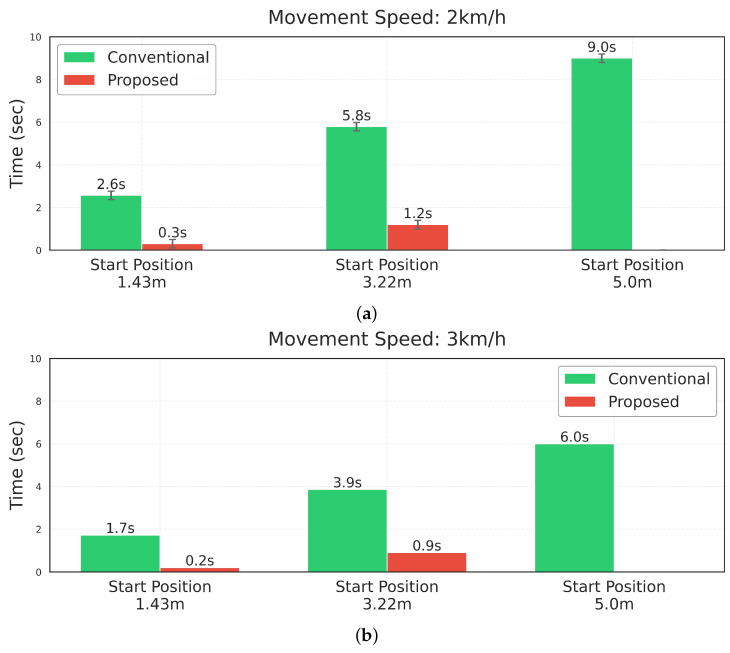
Comparison of collision detection activation time between conventional and proposed methods at different speeds of mobile robot.

Figure 26 demonstrates the comparison between conventional LiDAR-based detection and the proposed prediction-based detection algorithm. While the conventional method activates collision detection whenever obstacles enter the predetermined sensor range, our proposed method predicts the trajectory of moving obstacles and calculates potential collision points. This enables more precise detection timing and selective activation of the collision detection algorithm. As shown in the results, the proposed method significantly reduces unnecessary detection time compared to the conventional method (by up to 83% at 3 km/h), indicating its potential to minimize unnecessary speed adjustments while maintaining safety. This improved efficiency is particularly notable as the starting position of the moving obstacle increases. Additionally, in cases where no collision is predicted to occur (as shown in the case of 5.0 m starting position at 2 km/h), the proposed algorithm does not activate at all, further demonstrating its efficiency in avoiding unnecessary detection processes.

This feature is particularly crucial in indoor environments. Unlike road systems for vehicles, indoor spaces lack standardized traffic regulations, making it challenging to define clear behavioral guidelines or codes of conduct for robots. Consequently, representing interactions between robots and people in these environments is complex. Our algorithm addresses this challenge by providing a dynamic, time-based approach to human–robot interaction.

## 5. Discussion and Conclusions

As transportation costs have increased over the years, especially in last-mile delivery, many researchers are striving to reduce these costs by utilizing mobile robots [30]. Numerous studies have been conducted on stabilizing 4WIS mobile robots’ driving performance and analyzing their kinematics and driving modes [34]. However, the use of appropriate driving modes for 4WIS in specific situations and environments remains understudied. The results of our experiments reveal several significant findings regarding the effectiveness of 4WIS mobile robots in indoor environments.

Our experimental results showed that Parallel mode outperforms Ackermann mode in human detection and stability, especially at higher speeds and during turns. This suggests that Parallel mode is well suited for navigating dynamic indoor environments with frequent and unpredictable human presence. Its superior human detection and tracking capabilities make it particularly valuable for indoor applications where safety and reliability are paramount. The implemented time-to-collision prediction system represents a significant advancement in collision detection for mobile robots in human-shared environments. The algorithm’s ability to detect potential collisions and calculate the remaining time until a collision occurs enables greater operational efficiency based on human trajectory patterns. The system demonstrated up to an 83% reduction in unnecessary detection time compared to conventional methods at 3km/h, with improved efficiency particularly evident as the starting position of moving obstacles increased. This enhancement allows the robot to maintain efficient operations while ensuring safety in shared spaces.

However, several limitations need to be addressed in future work. In contrast to outdoor environments, where vehicles interact with pedestrians using standardized traffic rules and indicators such as turn signals and tail lamps, mobile robots lack such conventional communication methods [56,57]. In indoor settings, the absence of standardized traffic rules further complicates the establishment of consistent behavioral guidelines. Our time-based approach offers a flexible solution for defining robot behavior based on the remaining time to potential collisions, but further refinement is necessary. Unpredictable human movement patterns in indoor spaces pose significant challenges for collision prediction [45,63]. Current models may require further refinement to accommodate diverse human behavioral patterns and complex scenarios. Developing more sophisticated and context-aware interaction models will be crucial for enhancing the system’s effectiveness in real-world applications.

Future work should focus on extending our current dynamic path adjustment system in several ways. While our research has established the foundation for leveraging human trajectory data, there are opportunities to enhance the system’s sophistication and adaptability. Specifically, future research could explore the following:

1. Integration of machine learning approaches to improve the prediction accuracy of human movement patterns in complex scenarios [62];
2. Development of more sophisticated behavioral models that can account for group dynamics and social interactions in crowded indoor spaces [63,64,73].

These advancements would build upon our current time-based collision prediction framework while addressing the unique challenges of indoor human–robot interaction.

## Figures and Tables

**Table 1:** Steering angle criteria based on control signal format and wheel quadrant.

Linear.x	Linear.y	Quadrant	Direction	Steering Angle (*q*)
+	+	1	Front	+
+	−	2	Front	−
−	+	3	Rear	+
−	−	4	Rear	−

**Table 2:** The specifications of the 4WIS mobile robot.

Size	738 mm × 500 mm × 338 mm
Wheelbase	494 mm
Tread	364 mm
Ground Clearance	107 mm
Wheel Hub Radius	100 mm
Brake Type	Electronic brake
Suspension Type	Swing arm suspension

**Table 3:** Experimental specifications and parameters.

LiDAR channel/sampling rate	16 ch, 10 hz
Human walking speed	2 km/h
Robot velocity	0∼3 km/h
HD map resolution	50 mm/pixel

## Data Availability

The data presented in this study are available on request from the corresponding author. The data are not publicly available due to privacy restrictions, as they contain sensitive experimental information involving human interaction and specific proprietary algorithms used for collision detection and trajectory prediction.

## Supplementary Materials

The following supporting information can be downloaded at: https://drive.google.com/file/d/1w0vg-pFqLHeoHLKDXLTVhG0Q2xO4u6E4/view?usp=sharing, accessed on 9 January 2025. **Video S1**: Demonstration of the proposed 4WIS collision detection method in a human-shared indoor environment.
